# The Individualized Management Approach for Acute Poisoning

**DOI:** 10.1155/2021/9926682

**Published:** 2021-05-12

**Authors:** Muneera Al-Jelaify, Suliman AlHomidah

**Affiliations:** ^1^Pharmacy Services, King Khalid University Hospital, King Saud University Medical City, Riyadh, Saudi Arabia; ^2^Pediatric Intensive Care Unit, Pediatrics Department, King Khalid University Hospital, King Saud University Medical City, Riyadh, Saudi Arabia

## Abstract

Acute poisoning is a widespread emergency that mandates early management decisions for optimal outcomes. An individualized approach is an ideal way to provide those outcomes. Promoting awareness among healthcare professionals managing acute poisoning about the importance of incorporating the pharmacokinetics and following certain criteria to consider interventions such as activated charcoal, antidote, or specific investigations may improve their risk assessment strategies and management plans. To address the main aspects that should be considered to develop a customized poisoning management plan, we conducted this review based on relevant publications recovered by a careful search in PubMed. Our opinions as experts from the King Saud University (KSU) Drug and Poison Information Center (DPIC) were considered in the review.

## 1. Introduction

Acute poisoning is a widespread emergency situation that mandates early management decisions to safeguard optimal outcomes while at the same time avoids redundant investigation, intervention, or observation. An organized, individualized approach for patient assessment and management is the ideal way to provide the best emergency care for acute poisoning. The objective of this article is to address the main aspects that should be considered to develop a customized care plan for every patient [1].

The heterogeneous nature of patients presented with acute poisoning requires more than an understanding of the ingested substance. To design a rational therapeutic plan, many factors need to be considered: the ingested amount, time since ingestion, clinical presentation, patient factors, geographical site, and available resources. A patient-tailored approach is vital to ensure the best outcomes [1].

## 2. Our Poisoning Center and Experience in Poisoning Management

The Pharmacy in King Khalid University Hospital (KKUH) at King Saud University in the Riyadh area at the Kingdom of Saudi Arabia established the drug and poison information center in October 1983. The main objective to include poison information service was to provide evidence-based, expert advice from clinical pharmacists trained and certified to manage poisoning cases throughout the Kingdom of Saudi Arabia. The service, since its inception until present, offers consultations 24 hours (calls from 7: 30 am to 4: 00 pm are managed by the DPIC, while after working hours, consultations are managed by on-call clinical pharmacists). The provided information is targeted towards physicians, eventhough the advice was also available to other paramedical personnel and the common public [2].

Our center is experienced in providing optimal, patient-specific care that consider multiple factors. Such that, for any poisoning case regardless of the exposure route or the substance ingested, the general approach for management should be followed, which targets four main areas: initial assessment of airway, breathing and circulation, gastrointestinal decontamination, enhanced elimination, and antidotes. In addition to other specific factors that influence the therapy plan which include the following.

## 3. Drug Pharmacokinetics (PKs) in Overdoes [3–17]

We should always keep in mind that PKs in cases of overdose differ from the usual PKs, so the assumption that the usual PKs interpretation can be utilized for poisoning cases should be carefully implemented, especially for the majority of poisons; the kinetics during toxicity is rarely reported.

### 3.1. Onset of Action

Onset of action refers to the time required for a drug/agent response to be observed. It can aid to know if the poison action started. So one can anticipate the toxicity signs and symptoms and if antidote administration is considered, which is the best time to administer it, and when its action will start if given.

### 3.2. Time to Peak

Time to peak is defined as the time it takes for a drug/agent to reach its highest blood concentration. Although there is a rough relationship between the peak concentration of the poison and the occurrence of clinical toxicity, however, time to peak can still be utilized to predict a time window for appearance of signs and symptoms and help to decide about observation time and disposition, especially for drugs with long half-lives.

### 3.3. Bioavailability

Bioavailability refers to the fraction of the dose that reaches the systemic circulation. It can be affected by increasing the dose because of gastrointestinal (GI) physiology changes, saturation of absorption, or due to the first-pass effect. It can be determined by using the area under the curve (AUC) that presents the systemic exposure to the toxin. It cannot be estimated accurately in poisoning cases.

### 3.4. Half-Life

Half-life is the time it takes for the concentration of the drug/agent in the blood to be reduced by 50%. It can help to predict the toxic level of the drug/agent and help to decide about observation time and disposition and about the appropriate time for drug/agent levels or other lab tests.

It should not be used solely; rather, other parameters must be considered to decide on management since absorption, distribution, and clearance can change during an overdose. Also, the onset and duration of action in toxic doses may associate poorly with the half-life. It is very commonly seen that pharmacodynamics rely on distribution kinetics rather than clearance.

### 3.5. Volume of Distribution (Vd)

Volume of distribution reflects the distribution of a drug between plasma and the rest of the body. It has an adjunct role in poisoning management where you can use it to decide about dialysis as a toxin's elimination method since drugs with low Vd are more likely to be dialyzed.

You can also change the Vd by adjusting the fluid status to decrease the concentration in the blood if the ingested substance has a distribution limited to the plasma compartment. Also, it may aid in deciding about observation time and disposition. Developers of the drug nomograms such as paracetamol nomogram estimated the proper time to withdraw the drug level to be after 4 hours, allowing enough time for the drug to be absorbed and then distributed.

Poisonous substances undergoing redistribution or having a large volume of distribution may induce delayed toxicity manifestations because they will take a longer time to be eliminated even when using enhanced elimination strategies because those methods cannot reach through if the poison accumulated in the brain or lung, for example.

### 3.6. Protein Binding

Protein binding is the degree to which a drug attaches to plasma proteins (albumin, lipoprotein, and glycoprotein). It can magnify the severity of toxicity in the case of hypoalbuminemia for highly albumin‐bound substances, since hypoalbuminemia increases the free fraction of the drug in the blood, which increases the risk of toxicity, and can give a clue about the possibility for the ingested substance to undergo dialysis or plasma exchange, where drugs with low protein binding affinity are more likely to be dialyzed, while drugs with high protein binding affinity are more likely to be removed by plasma exchange.

### 3.7. Metabolism

Define first if the drug is metabolized by the liver or excreted mostly unchanged in the urine. If metabolized, then check whether the metabolites are active, as it will extend the duration of toxicity. Also, if metabolism will reach saturation, it can result in unpredictable toxin's concentration.

Reduction in the first-pass effect can dramatically increase the absorption of oral drugs even with therapeutic doses like in patients with cirrhosis. This change will make the dose and toxicity relationship unpredictable for drugs undergoing significant first-pass effects (e.g., calcium channel blockers).

Using the enzyme induction method to enhance poison elimination is a theory that cannot be utilized in management of acute poisoning where care should be immediate while enzyme induction may take time to work. But reducing the amount of toxic metabolites can help, such as in cases of alcohol poisoning.

### 3.8. Excretion

Check first if the poison is eliminated mainly by the kidney in urine or not. If so, the half-life will be affected. This will affect the decisions to utilize enhanced elimination methods, including dialysis or urine alkalinization for weak acid drugs and the observation/disposition time. The longer the half-life, the longer the time the victims may need under observation.

### 3.9. Pharmacokinetics Role in Dialysis Decision

Knowing the PKs and physicochemical characters of the ingested drug will help decide the applicability of dialysis as a toxin elimination method, if the drug has a small molecular weight and volume of distribution, a low affinity of binding to plasma proteins, and a low endogenous rate of clearance, then the toxin is considered dialyzable.  Table 1 presents some of the characteristics with the most applicable removal method [4, 18].

Common examples of drugs where toxicity can be severe and dialysis options are considered a therapeutic solution are carbamazepine and phenytoin.  Table 2 summarizes the PKs properties of each drug and the likeliness of dialysis efficacy [19–21].

## 4. Activated Charcoal (AC) [22–24]

AC is still considered a universal antidote for acutely poisoned patients, eventhough its use has declined significantly in recent years. This is mainly attributed to it is possible risks, uncertainty about its benefits [25–28], and that clinicians have less interest in GI decontamination as a mode of therapy. The commonly mentioned safety concern is a pulmonary aspiration, but the incidence is still low and increases with risk factors such as vomiting, seizures, and altered mental status. Some very rare adverse events from case reports include GI complications (emesis, esophageal perforation, GI perforation, and intestinal obstruction), pulmonary complications (chronic lung disease, obstructive laryngitis with glottic edema, granulomatous lung mass, charcoal empyema, and bronchiolitis obliterans), and corneal abrasions.

### 4.1. Usual Conditions and Requirements to Consider Single-Dose AC

  The ingested agent can be adsorbed to AC
  Severe toxicity is expected
  Recent overdose
  Conscious and alert patient
  Secured airway
  The antidote is not available
  Ingestion of long-acting agents (i.e., modified, extended, or sustained dosage forms)
  No intestinal obstruction or ileus

### 4.2. Timing of Administration

Several studies concluded that the optimal time for administration of AC is within 1 hour of poison ingestion. However, late administration beyond 1 hour can be considered in some cases, for example, if a large quantity of an agent is administered, which carries a very high risk of morbidity and mortality with very limited therapeutic options. Moreover, it can be used when there is a high chance that the ingested drug/agent remains in the GI tract (i.e., coingestion of drugs that slow gastric emptying or in the presence of food), and using AC might positively impact the patient's outcomes.

This mnemonic (PHAILS) outlines the common agents and conditions for which AC is not generally indicated [22, 25, 29–33]:

 
**P**—**P**esticides, **P**etroleum distillates, un-**P**rotected airway
 
**H**—**H**ydrocarbons, **H**eavy metals, >1 **H**our presentation
 
**A**—**A**cids, **A**lkali, **A**lcohols, **A**ltered level of consciousness, **A**spiration risk
  I—**I**ron, **I**leus, **I**ntestinal obstruction
 
**L**—**L**ithium, **L**ack of gag reflex
 
**S**—**S**olvents, **S**eizures

## 5. Antidotes, Clinical Pearls [34]

  Value from antidotes is generally time-dependent without certainties. For many victims of poisons, clinical improvement is expected with supportive management only. Most of the poisoning-related mortalities are because of a lack of timely supportive management rather than the absence of antidote administration. Despite the enthusiasm by clinicians to use antidotes, the approved antidotes are around 20 that cannot cover the huge number of drugs and toxins available.
  For ideal antidotes use, recognize conditions in which their use could yield clinically significant improvements in morbidity or mortality. If the utility cannot be quantified, estimate the risk from giving the antidote. Many common antidotes are primarily safe (e.g., activated charcoal or vitamins), while some can cause hypersensitivity reactions (e.g., N-acetylcysteine or antivenoms); dosing errors is another risk factor that limits antidote use (e.g., an overdose of flumazenil or naloxone can induce seizures). The toxicity from using antidotes can be precipitated or exaggerated when administered for multiple overdoses (e.g., flumazenil may provoke seizures without recovery from coma in patients with a concomitant antipsychotic overdose).

## 6. Investigations and Screening Tests [1, 3]

The most commonly recommended screening tests for acute poisoning are ECG and the serum paracetamol level. Drug concentrations are required only for few agents for the purpose of risk assessment and to decide on timely needed interventions (e.g., paracetamol, salicylate, and iron). Other investigations are requested selectively where the findings support management.

Some of the compelling indications for specific tests in acutely poisoned patients may include the following:

  Guide risk assessment or prognosis
  Help to include or exclude differential diagnoses, complications, and suspected poisonous agents
  Help to decide about the benefit of decontamination methods and/or antidote administration
  Monitor the response to provided care and the limitations or endpoint to ongoing interventions

## 7. Observation and Admission Criteria [3, 17]

Judgments related to hospitalization for patients with suspected poisoning are occasionally difficult. The majority of poisoning victims will be asymptomatic, and a short observation time in the emergency room is often the only necessary thing to do.

The type (drug, household products, etc.), nature (e.g., acidic or basic, lipid base or hydrophilic), amount, form (e.g., immediate-release or sustained), pharmacokinetics, and pharmacodynamics of the ingested substance should always be taken into consideration, as it will be very helpful to decide about disposition or admission and the required time for observation and monitoring.

It is very important during the formulation of the caring plan to investigate the nature and history of the poisoning case. Intentional overdose may indicate psychosocial troubles, while an unreliable history in children may point to the likelihood of abuse or neglect. In both situations, you need to admit the child for expert consultations.

## 8. Conclusion

An individualized management approach is an optimal way to provide patient care. Promoting awareness among healthcare professionals managing acute poisoning about the importance of incorporating the pharmacokinetics parameters and following certain criteria to consider interventions such as activated charcoal, antidote, or specific investigations or lab tests may improve their risk assessment strategies and management plans.

## Figures and Tables

**Table 1:** Drug characteristics and the corresponding suitable dialysis method.

	Hemodialysis	Hemoperfusion	Hemofiltration
Molecular weight	<500 Da	<50,000 Da	<50,000 Da
Protein binding	Poorly bound	Low or high	Poorly bound
Volume of distribution	<1 L/Kg	<1 L/Kg	<1 L/Kg
Solubility	Hydrophilic	Hydrophilic or lipophilic	Hydrophilic
Endogenous clearance	<4 mL/Kg/min	<4 mL/Kg/min	<4 mL/Kg/min

**Table 2:** Carbamazepine and phenytoin PKs and the likeliness of dialysis efficacy.

Drug	Usual PKs	PKs in overdose	Efficacy of dialysis
Carbamazepine	(i) Molecular mass: 236 Da (small) (ii) Vd: 2 L/kg (high); lipophilic (iii) Protein binding: 70–80% (high) (iv) Initial half-life is 25–65 h and then 10 h (v) Active metabolites (vi) Urine elimination (72%) (vii) Induces its own metabolism (chronic use) (viii) Time to peak = 4 hours	(i) Protein binding does not decrease significantly in overdose (ii) No enzyme induction in CBZ naive individuals (iii) Half-life much longer (ongoing absorption, impaired elimination, or both) (iv) High concentration exhibits anticholinergic proprieties, which delay GI motility, further prolonging absorption, with time to peak > 100 hours	High-efficiency hemodialysis is the best method Then, venovenous CRRT and charcoal or resin hemoperfusion May reduce the carbamazepine level by about 50%

Phenytoin	(i) Molecular mass: 252 Da (low) (ii) Vd: 0.7 L/kg (low) (iii) Protein binding: 90–95% (high) (iv) Half-life = 22 h (v) Cl = 23 mL/min (vi) Follows nonlinear PKs	(i) Clearance reduced (ii) Half-life greatly prolonged (103 hours) (iii) Protein binding decreases (70–80%) (iv) Volume of distribution increases	High-efficiency hemodialysis is the best method, but hemoperfusion is an acceptable alternative. However, because of the low incidence of irreversible tissue injury or death related to phenytoin poisoning and the relatively limited effect of dialysis on phenytoin removal, dialysis can be used only in very selected patients with severe phenytoin poisoning.
